# Genomic Indexing by Somatic Gene Recombination of mRNA/ncRNA – Does It Play a Role in Genomic Mosaicism, Memory Formation, and Alzheimer’s Disease?

**DOI:** 10.3389/fgene.2020.00370

**Published:** 2020-04-29

**Authors:** Uwe Ueberham, Thomas Arendt

**Affiliations:** Paul Flechsig Institute for Brain Research, University of Leipzig, Leipzig, Germany

**Keywords:** Alzheimer’s disease, LINE-1 (L1), mosaicism, neuronal individuality, human brain, amyloid precursor protein (APP), retrotransposition, somatic gene recombination

## Abstract

Recent evidence indicates that genomic individuality of neurons, characterized by DNA-content variation, is a common if not universal phenomenon in the human brain that occurs naturally but can also show aberrancies that have been linked to the pathomechanism of Alzheimer’s disease and related neurodegenerative disorders. Etiologically, this genomic mosaic has been suggested to arise from defects of cell cycle regulation that may occur either during brain development or in the mature brain after terminal differentiation of neurons. Here, we aim to draw attention towards another mechanism that can give rise to genomic individuality of neurons, with far-reaching consequences. This mechanism has its origin in the transcriptome rather than in replication defects of the genome, i.e., somatic gene recombination of RNA. We continue to develop the concept that somatic gene recombination of RNA provides a physiological process that, through integration of intronless mRNA/ncRNA into the genome, allows a particular functional state at the level of the individual neuron to be indexed. By insertion of defined RNAs in a somatic recombination process, the presence of specific mRNA transcripts within a definite temporal context can be “frozen” and can serve as an index that can be recalled at any later point in time. This allows information related to a specific neuronal state of differentiation and/or activity relevant to a memory trace to be fixed. We suggest that this process is used throughout the lifetime of each neuron and might have both advantageous and deleterious consequences.

## The Individuality of Neurons

The individuality of neurons provides an accepted paradigmatic framework for the nervous systems of invertebrates such as Caenorhabditis elegans or Drosophila melanogaster, where the uniqueness of each single neuron is reflected by its particular spatial and functional position (Alicea, 2018; Davie et al., 2018; Hammarlund et al., 2018). With current technical developments that allow for high-throughput analyses of various cellular markers, it is becoming more and more obvious, however, that in vertebrates, too, and even in primates, neurons are much more heterogeneous than previously thought (Lake et al., 2016; Lim et al., 2018). Thus, depending on how many parameters can be assessed in depth simultaneously, each neuron might be unique with respect to its functional, structural, and molecular signatures. This cellular diversity and individuality might result from a complex process where different determinants interacting at different levels, such as developmental trajectories, relationships with neighboring cells, functional integration in neuronal networks, and others, may shape and re-shape cellular signatures.

One particular aspect that is crucial to our understanding of the individuality of somatic cells is the individual genetic equipment giving rise to genetic mosaicism. Commonly, mosaicism is defined as the presence of genetically different lineages of cells derived from a single zygote, with additional variations arising in the soma of each cell that are usually not inherited by the next generation (Forsberg et al., 2017). Genomic mosaicism in the human brain has been explored for about 20 years (Muotri et al., 2005; Renthal et al., 2018; Saleh et al., 2019) and is currently a subject of intensive research (see this special issue). While the phenomenon of genomic mosaicism now seems to be an established fact (Rohrback et al., 2018b), there is much less consensus on its extent and distribution (Paquola et al., 2017; Rohrback et al., 2018a), and hardly anything is known about its physiological and potentially pathophysiological meaning.

Research on single-neuronal DNA content in human brain over the last 20 years or so has collected a huge but, to some extent, inconsistent pool of data. Searching for cellular signatures of neurodegenerative diseases such as Alzheimer’s disease (AD) was a particular driving force for early studies (Potter, 1991; Potter and Geller, 1996; Geller and Potter, 1999; Rehen et al., 2001; Mosch et al., 2006, 2007; Arendt et al., 2009, 2015, 2017; Arendt, 2009; Fischer et al., 2012).

First reports, based on analyses of bulk DNA, derived from a mixture containing neuronal and non-neuronal cells of the human brain, showed alterations of DNA content (Rehen et al., 2005). Subsequent studies, applying more sophisticated techniques of single-cell analyses based on single-cell isolation by high-throughput cell sorting or laser capture microdissection in combination with single-cell sequencing, identified chromosomal aneuploidy, small and larger copy number variations (CNVs), single nucleotide variations (SNVs), and DNA content variation (DCV), all contributing to the genomic heterogeneity and individuality of each single neuron (Mosch et al., 2006, 2007; Arendt et al., 2009; Iourov et al., 2009; Westra et al., 2010; Fischer et al., 2012; Abdallah et al., 2013; McConnell et al., 2013). Excellent reviews on this phenomenon and the underlying mechanisms are provided in this special issue and elsewhere (McConnell et al., 2017; Rohrback et al., 2018b).

## A Very Large Part of the Human Genome Might Be of Retro-Transposable Origin

In a comparison of the predicted number of protein-coding genes in a wide range of phylogenetically related vertebrates, only 16,000–26,000 hits are proposed (Holland et al., 2017). Most of them comprise complex exon-intron structures allowing the splicing machinery to generate transcripts in a cell-type- and time-dependent context. However, for the human genome, protein-coding transcripts cover only about 2%, whereas 75% of the human genome can be transcribed (Djebali et al., 2012) and are currently attributed to the ncRNA fraction. In this context, it is important to note that more than 40% of DNA sequences are assumed to be of retro-transposable origin (Cordaux and Batzer, 2009). The function of these sequences is still mostly unknown but is receiving increasing amounts of attention, especially with respect to unveiling the heterogeneity of single cells in selected tissues, particularly in the brain.

Most approaches to assess a potential function of somatic genomic mosaicisms in both health and disease largely ignore the role of RNA or, at the most, attribute to RNA only a canonical function within the context of transcription and translation of genetic information. There are, however, a few most intriguing studies suggesting a role for RNA in DNA sequence modulation, e.g., transcription-associated mutagenesis or transcription-associated recombination resulting from events like RNA collision with replication machinery or co-transcriptional R-loop formation (Green et al., 2003; Majewski, 2003; Polak and Arndt, 2008; Kim and Jinks-Robertson, 2012; Garcia-Muse and Aguilera, 2019; Rondon and Aguilera, 2019). While immunoglobulin class-switch recombination, which generates diverse antibodies, is a beneficial example of R-loop formation (Yu et al., 2003), in several repeat-associated neurological diseases, such RNA-DNA-hybrids produce deleterious DNA sequence modifications (e.g., RNAs from HTT, FXN, or ATXN1) (Richard and Manley, 2017; Neil et al., 2018).

Recently, a study by Lee et al. (2018) reported on the discovery of somatic gene recombination in terminally differentiated human neurons. They identified thousands of variant genomic cDNAs (gencDNA) of the amyloid precursor protein (APP) gene in neurons of Alzheimer’s disease brains. These gencDNAs contained no introns but showed a wide range of sequence pattern comprising full-length copies of brain-specific splice variants and many smaller forms with insertions, deletions, single-nucleotide variations, or intra-exonic junctions. According to their data, a “retro-insertion” of RNA is a likely source of these gencDNAs. Though highly enriched in the neurons of AD brains, where several known and some unknown APP mutations could be identified, control brains also showed gencDNA loci of recombined APP. Apparently, genomic recombination is a common process in terminally differentiated neurons in both normal and diseased brain, contributing to mosaicism, individuality, and pathology.

## Intronless Genes

A striking observation by Lee et al. (2018) is the detection of thousands of intronless APP-derived sequences in the DNA of single neurons. Though data on further intronless genes of a comparable extent have not yet been reported, the questions arise: what are the possible reasons for the usage of intronless transcripts, and do they fulfill a physiological function?

Firstly, introns are a characteristic feature of eukaryotic genomes. They are genetic elements that can monitor their own gene transcription or the transcription of functionally clustered genes (Hube and Francastel, 2015; Shaul, 2017). Following this idea, a feedback control could avoid unnecessary accumulation of toxic metabolites or proteins to protect cells and to avoid energy/substrate wastage. The presence of introns can thus contribute to better regulation of the genome and increases its coding potential (Heyn et al., 2015). They also provide a mechanism to increase the proteome diversity by alternative splicing (Nilsen and Graveley, 2010). Introns can protect eukaryotic genomes from transcription-associated genetic instability, for example by preventing R-loop formation and DNA damage accumulation (Bonnet et al., 2017). However, a remarkable fraction of constitutively spliced transcripts using the intronic gene structure that might not contribute to substantial regulation has also been identified (Ryu et al., 2015). Accordingly, constitutive exons are evolutionarily older, and their replacement by alternative exons has only restricted functional relevance (Xiong et al., 2018), suggesting a possible role for basic cellular functions. Thus, constitutive exons behave at least partly like intronless transcripts. Of note, several housekeeping genes such as GAPDH or ACTH, the expression of which is assumed to be relatively stable within cells, possess a high number of mostly intronless pseudogenes, comparable in size to their authentic RNA (Sun et al., 2012).

## Intronless Genes Can Contribute to the Genomic Diversity of Cells

Up to 10% of sequences that appear as pseudogenes in the human genome seem to be transcribed (Djebali et al., 2012) and could participate in gene expression as a competing endogenous RNA (ceRNA) (Poliseno, 2012; Zhong et al., 2018) or might even code for translated protein (Ingolia et al., 2014; Ji et al., 2015).

Intronless genes, which represent less than 5% of the human genome, lack intron-dependent transcription control, leading to a more constant expression level. Such features have been reported for genes that preferably encode metabolically passive proteins (Hill and Sorscher, 2006).

Most intronless genes are evolutionarily relatively young, are expressed at lower levels compared to intron-containing genes, show a higher tissue specificity, and evolve faster than spliced genes (Shabalina et al., 2010; Louhichi et al., 2011). It seems that intronlessness is a more recent form of evolution to develop tissue-specific functions (Brosius and Gould, 1992; Shabalina et al., 2010) that might be actively involved in brain development and aging.

An unusually high number of intronless genes have neuron-specific expression (Grzybowska, 2012) or at least play a major role in the brain, such as several serotonin receptors, HTR1A, HTR1B, or HTR1D or beta1- and beta2-adrenergic receptors (ADRB1, ADRB2) (see the IGD database^1^; Louhichi et al., 2011).

Intronless transcripts circumvent the complex splicing process, thereby saving energy and time and allowing for replication of much shorter genes. Splicing mechanisms always pose a definite risk of inaccurate execution. Thus, a globally impaired exon exclusion and selective loss of splicing factors have been shown for AD brains (Berson et al., 2012). Moreover, destruction of cholinergic neurons in mice, a critical feature of AD pathology, leads to disturbances in RNA splicing, dendritic loss, and memory impairment (Kolisnyk et al., 2016). During aging, the number of splicing errors increases in the brain. Recently, an integrative transcriptome analysis of the aging brain provided evidence that deregulated mRNA splicing is a feature in AD, where hundreds of aberrant pre-splicing events could be detected (Raj et al., 2018).

The number of somatic mutations in the human brain increases over the lifetime due to various types of stress and an age-related loss of DNA repair efficiency, which itself is comprised of mutations contributing to this genomic instability (Chow and Herrup, 2015; Verheijen et al., 2018). Usage of such compromised DNA could be prevented by the availability of alternatively saved/stored intronless variants. RNA molecules could serve as templates to repair DNA double-strand breaks leading to intronless genes (Catania, 2017).

Taken together, several lines of evidence suggest that intronless genes, which, to some extent, may appear as pseudogenes, could substantially contribute to the genetic diversity of cells (Kovalenko and Patrushev, 2018).

## Intronless Genes Are a Likely Consequence of Somatically Recombined Transcript Incorporation Into the Genome and Are Potentially Generated by Line-1 Retrotransposition

A likely source of intronless genes in eukaryotic genomes is the retroposition of cellular mRNAs by retrotransposable elements (Callinan and Batzer, 2006; Baertsch et al., 2008), though Lee et al. (2018) could not confirm this for APP-derived intronless transcripts. However, during the evolution of the primate lineage, there was a burst of retropositions that reached its peak about 38–50 million years ago, when many intronless genes emerged in the genome (Marques et al., 2005).

Retrotransposons are mobile elements that account for more than 40% of the human genome (Lander et al., 2001). They have been identified as an important source for genetic variations during the evolution of the human genome. However, only a limited number of these elements retain full function and are still active in the genome. The vast majority of retrotransposons are silenced at multiple levels, including transcriptional repression, epigenetic modification such as DNA methylation of CpG-rich promoters (Greenberg and Bourc’his, 2019), or other post-transcriptional gene-regulation mechanisms.

The evolutionary bursts of retrotransposable elements in the human genome gave rise to about 700 human intronless genes, which stabilized biological processes critically required for the survival of the species (Louhichi et al., 2011). Generation of intronless genes through retrotransposable elements can apparently take place both in the germline and somatic cells. Thus, many intronless genes are inherited and, accordingly, show testis-specific expression (Grzybowska, 2012). In addition, during the lifetime of individual organisms, retrotransposable elements might give rise to intronless genes in somatic cells such as neurons, where they could contribute to the genomic individuality of neurons as well as to the individuality of the carrier organism.

The long interspersed element (L1, LINE-1) is the only known active autonomous retrotransposon in human cells (Moran et al., 1996) and covers up to 17% of the human genome. About 100 retrotransposition-competent L1-elements are detected in each individual, while more than 500,000 copies are silent due to truncations, deletions, or other alterations (Myers et al., 2002; Brouha et al., 2003; Salvador-Palomeque et al., 2019). The view of L1 elements has changed over time from being regarded as “selfish” or “parasitic” towards representing functionally critical elements (Paco et al., 2015) that fulfill essential roles in the regulation of gene expression. However, addressing the function of LINE-1 elements has been restricted by technical difficulties in detecting their specific location in the human genome. Their high copy number often gives rise to unreliable data in PCR amplifications or hybridization-based assays, and new methods for mapping active transposable element insertion sites in genomic DNA have been developed only recently (Steranka et al., 2019).

**FIGURE 1:**
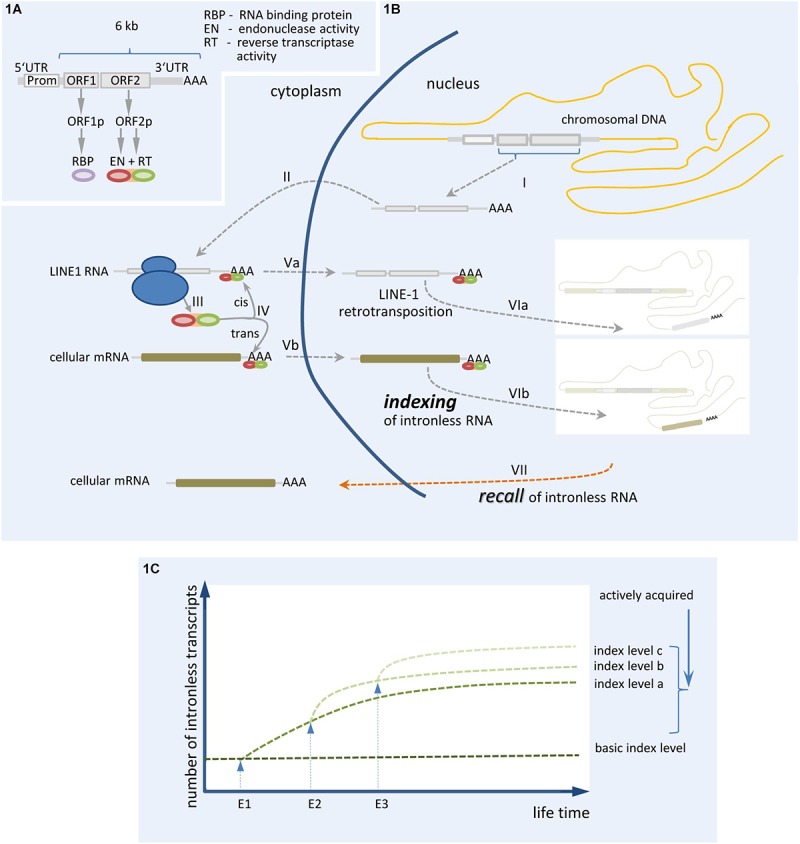
Synopsis of the proposed mechanism of genomic indexing by somatic gene recombination of mRNA/ncRNA. **(A)** The restrotransposition competent (RC) LINE-1 RNA and the encoded proteins are shown. **(B)** The process of LINE-1 directed retrotransposition and genomic indexing by somatic gene recombination of mRNA is depicted: (I) transcription of retrotransposition competent (RC) LINE-1 controlled by endogenous promoter, (II) transport of RC-LINE-1 transcript to cytoplasm, (III) translation of ORF1 and ORF2 proteins, (IV) binding of ORF2 protein (and ORF1 protein, not shown) to their own mRNA (cis) or a cellular mRNA (trans) (potentially representing a specific cellular context) by forming a ribonucleoprotein complex, (Va/Vb) transport of cis- or trans-generated ribonucleoprotein complex into the nucleus, (VIa/b) retrotransposition is controlled by Target Primed Reverse Transcription (TPRT) in “VIb,” leading to indexing of a specific cellular context, and (VII) recall of intronless RNA. **(C)** Proposed operational sequence leading to an increasing genomic index or memory trace by somatic gene recombination. E1, E2, and E3 represent events leading to increased index levels due to the insertion of RNA transcripts (generated within a definite temporal context) by somatic recombination. Whether single events finally provide advantageous or deleterious indices depends both on the spatial/temporal context and whether the RNA transcripts used for genomic recombination correspond to a correct or a mutated sequence.

Members of the LINE-1 retrotransposon family typically use target-primed reverse transcription (TPRT) to generate *de novo* insertions into genomic locations of germline and somatic cells. TPRT is catalyzed in cis by ORF1p and ORF2p, two proteins translated from a bicistronic 6 kb L1 mRNA (Figure 1A). The L1 ORF2p comprises both endonuclease activity (EN) and reverse transcriptase (RT) activities, which are essential components for successful L1 retrotransposition (Mathias et al., 1991; Feng et al., 1996). Retrotransposition is started by an internal promoter located in the L1 5′-untranslated region (Swergold, 1990). Synthesized L1 mRNA is subsequently transported to the cytoplasm (Figure 1B), where ORF1p and ORF2p proteins are translated and bind their own mRNA to form a ribonucleoprotein particle (Wei et al., 2001). After entering the nucleus, TPRT activity catalyzes the retrotransposition (Upton et al., 2015). Intragenic insertions of LINEs can disrupt gene expression, which is often connected to severe diseases (Schwahn et al., 1998; Meischl et al., 2000). Recently, LINE elements have been inferred to participate in recruiting RNA-binding proteins to mammalian introns and to influence the splicing and evolution of tissue-specific exons (Attig et al., 2018). The ability of evolutionarily young LINEs to attract splice-repressive RNA binding proteins (e.g., MATR3, PTBP1) contrasts with evolutionarily old LINEs, which possess less repressive motifs but rather allow for the binding of splice-promoting RNA-binding proteins. These latter LINEs support lineage-specific splicing (Attig et al., 2018) and play an important role in the development of neurons, making the brain a hotspot of somatic mosaicism. Apparently, L1 mobilization operates during the entire life-span of neurons, starting during neurogenesis in neuronal precursor cells (NPC) (Muotri et al., 2005, 2009; Coufal et al., 2011; Upton et al., 2011; Kurnosov et al., 2015; Macia et al., 2017) and persisting into terminally differentiated states (Baillie et al., 2011; Evrony et al., 2012, 2015; Erwin et al., 2016).

Relevant to the above-mentioned generation of somatically recombined transcripts is the ability of LINE-1 transcripts to retrotranspose cellular mRNA in trans (Wei et al., 2001; Figure 1B). To this end, both intact ORF1p- and ORF2p-encoded proteins are necessary. Different data on the frequency of pseudogene formation, between 0.01 and 0.05% of the rate of L1 retrotransposition (Wei et al., 2001) and about 10% (Esnault et al., 2000), were reported and suggested different integration mechanisms with respect to L1-endonuclease (Wei et al., 2001). However, *in vitro* mature neurons express detectable L1 mRNA and ORF1p levels and exert efficiently engineered L1 retrotransposition (Macia et al., 2017).

## What Is Necessary for Somatic Recombination of RNA-Based Transcripts in Trans?

The mechanism of retro-insertion of RNA-based transcripts in trans has a number of prerequisites such as reverse transcriptase activity, poly-adenylation, and DNA double-strand break. The availability of these factors will determine the frequency and efficacy of retro-insertion.

Reverse transcriptase (RT) activity has recently been detected in normal human brain extracts (Lee et al., 2018) and blood samples (Steele et al., 2005; MacGowan et al., 2007). Reverse transcriptase activity seems phylogenetically of different origins, with non-LTR reverse transcriptase, including group II intron IEPs, telomerase, and human L1 reverse transcriptase, differing from LTR enzymes, which include the HIV enzymes (Zhao and Pyle, 2017). However, retrotransposons are potential sources for reverse transcriptase activity in human neurons. The family of human-specific LINE-1 retrotransposons is the only family known so far that can actively and autonomously transpose into the human genome, thereby using its own encoded protein activities necessary for retrotransposition (e.g., endonuclease and reverse transcriptase) (Kazazian and Moran, 2017).

Replacement studies demonstrated that a poly(A) sequence is required for LINE-1 directed retrotransposition (Doucet et al., 2015), where not only cis encoding L1 retrotransposons, which end in a 3′ poly(A) sequence, are mobilized, but also cellular mRNA in trans can be a target (Doucet et al., 2015).

The poly(A) tail of mRNA facilitates its export from the nucleus, enhances protein synthesis, and stabilizes mRNA by interacting with poly(A)-binding proteins to prevent exonucleolytic degradation.

Interestingly, it has been reported that non-conserved poly(A) sites are associated with transposable elements to a much greater extent than conserved ones (Lee et al., 2008). This opens the opportunity for LINE-1 elements to differently use alternative polyadenylation (APA) sites of individual mRNA transcripts, which influence mRNA stability, mRNA localization, or the amount and localization of encoded proteins (Tian and Manley, 2017). Especially for the brain, a wide variety of APA is known, which is typically associated with a particular expression pattern specific to a cell-type or even subcellular compartment (Miura et al., 2013; Taliaferro et al., 2016). For example, for brain-derived growth factor (BDNF), a short isoform of the mRNA is restricted to the cell body, whereas the long isoform localizes to the dendrites, where it is translated (An et al., 2008).

APAs could thus provide a broad basis for the incorporation of selected transcripts into the genome of each single neuron by somatic recombination according to their individual profile. Additionally, a potential LINE-1 insertion candidate RNA can possess poly(A) tails of different lengths, obtained by somatic mutation, which are finally reverse-transcribed into the genome (Evrony et al., 2015). Data from the same study indicated the existence of highly polymorphic poly(A) tails of varying length, leading to many different somatic mutations, which can contribute to manifestations of local and functional clones. This might also contribute to the highly diverse mosaicism observed in neurons.

While polyadenylation of RNA is required for labeling RNAs to prevent degradation processes, the primordial role of oligoadenylation is RNA tagging for subsequent destabilization, which blurs the boundary between stabilization and destabilization by adenylations (Tudek et al., 2018). It might thus be tempting to speculate that truncated mRNA transcripts, which are intended to be degraded and so are oligoadenylated, might be at risk of being accidently included in “normal polyadenylation processes.” This kind of potential RNA “mislabeling” might trigger accidental translation and protein synthesis or even lead to interaction with transposable elements such as LINE-1, which in turn allow the integration into the genome of individual cells and contribute to mosaicism. TENT2, also known as GLD2, a non-canonical poly(A) polymerase, is such a possible candidate, which; performs both polyadenylation and oligoadenylation on many RNAs (e.g., GluN2A RNA), is expressed in the hippocampus, can co-localize with proteins relevant for synaptic plasticity, and may be necessary for long-term potentiation (Rouhana et al., 2005; Swanger et al., 2013). Other non-canonical poly(A) polymerases, e.g., TENT4A/B are involved both in RNA decay and in the stabilization of mRNAs (Gagliardi and Dziembowski, 2018; Warkocki et al., 2018).

A further requirement for the generation of somatically recombined transcripts and their DNA integration are DNA double-strand breaks. Among others, DNA double-strand breaks have been linked to tumorigenesis and genetic instabilities (Aparicio et al., 2014; Mladenov et al., 2016). In addition, disturbances of the underlying repair mechanisms, which involves a coordinated action of TDP2 (tyrosyl DNA phosphodiesterase 2) with enzymes of the NHEJ repair pathway, can lead to neurological diseases associated with intellectual impairment or ataxia (Gomez-Herreros et al., 2014). Moreover, corruption of epigenetically modified DNA in the germline followed by errors in the subsequent repair process could even lead to epigenetic regulatory effects transmissible over generations as an epigenetic memory of repair of DNA double-strand breaks (Orlowski et al., 2011). While any insertion following DNA double-strand breaks can be mutagenic through disrupting coding sequences, it can also influence the expression of adjacent genes by reorganizing the gene structure, providing completely new features, and could therefore also be physiologically relevant.

## Line-1 Integration Is Involved in Memory Formation

Recently, it has been reported that DNA double-strand breaks linked to neuronal activity are a common, basic, and physiological phenomenon. Exploratory activity in mouse, for example, which is associated with increased neuronal activity, has been shown to cause a significant increase in neuronal DNA double-strand breaks (Suberbielle et al., 2013). Moreover, a variety of early-response genes, such as Fos, FosB, and Egr1, other transcription factors, such as Olig2, and ncRNAs, such as Malat1, are targets of activity-induced DNA double-strand breaks in neurons (Madabhushi et al., 2015).

LINE-1 mobilization in brain uses functionally active DNA double-strand breaks to jump into the genomic DNA. The linkage of DNA double-strand breaks to neuronal activity (Suberbielle et al., 2013) might thus provide a mechanism to index the specific activity state of the neurons.

L1 insertions in neurons were proposed to be a mechanism of “genomic plasticity” some years ago (Singer et al., 2010). Accordingly, L1 elements alter the neuronal transcriptome by their genomic integration, which eventually contributes to a modified behavior of the affected individual (Singer et al., 2010). Moreover, the involvement of LINE-1 activation in memory formation has recently been reported (Bachiller et al., 2017). Immediately after a novel place exploration session in mice, a short and temporarily limited increase of LINE-1 orf1- and orf2- mRNA expression was observed in the hippocampus. Remarkably, just 1 h after the exploratory session, a permanently elevated copy number of orf2- insertions in the hippocampal genome was measured, while the content of orf1 sequences did not change. The orf2 copy number increase in genomic DNA could be blocked by the administration of lamivudine, a retrotranscriptase inhibitor. Accordingly, lamivudine application within a time window of 6 h after the training session also impaired long-term memory formation. Both memory formation and orf2 insertion in DNA was also prevented by orf1 antisense or orf2 antisense RNA infusion into hippocampus (Bachiller et al., 2017; Wang et al., 2018). Another study (Kokaeva et al., 2002) has shown that inhibition of the expression of the LINE-1 reverse-transcriptase gene in rats by antisense oligonucleotides disturbed the formation of long-term memory, while short-term memory was not altered.

Taken together, there is thus strong evidence that L1 retrotransposon insertions might be involved in the process of long-term memory formation.

The majority of studies on the *de novo* genomic LINE-1 insertion has mainly focused on the alteration of mRNA/ncRNA expression levels or relocation of splicing-variant-ratios. However, the detection of hundreds of somatically recombined APP sequences in neurons of both the healthy and diseased human brain (Lee et al., 2018) together with accumulating evidence linking LINE-1 to memory formation, opens up a completely new perspective on the role of transcribed mRNA/ncRNA in indexing functional states of neurons.

We would thus like to further develop the hypothesis that somatic recombination of intronless mRNA/ncRNAs provides a mechanism to index a particular functional state at the level of the individual neuron, a suggestion that was similarly proposed by Lee et al. (2018). By insertion of defined RNAs in a somatic recombination process, the presence of specific mRNA transcripts within a definite temporal context could be “frozen” and serve as an index that can be recalled. This allows the fixing of information related to a specific neuronal state of differentiation and/or activity relevant to a memory trace. This process might take place throughout the lifetime of each neuron and will potentially have both advantageous and deleterious consequences (Figure 1C).

In conclusion, it might thus be probable that retrotransposition by LINE-1, which allows the use of defined RNA to index a particular cellular state, represents a powerful and versatile toolbox in somatic cells that can modify the DNA sequence without affecting original gene structures.

## Are Line-1 Activity and gencdna Generation Involved in the Pathomechanism of Ad?

While a definite involvement of LINE-1 and gencDNA in the AD pathomechanism remains to be shown, several lines of evidence clearly point in this direction. A recent analysis of more than 600 human cortical transcriptomes indeed revealed evidence for a global transcriptional activation of LINE-1 in AD (Guo et al., 2018). Still, another study analyzing only a small number of AD samples by target PCR failed to detect any differences in L1 genomic copy numbers (Protasova et al., 2017).

Global hypomethylation of DNA, accompanied by a downregulation of neuronal DNA methyltransferase 1 (DNMT1), appears to be a characteristic feature of AD (Mastroeni et al., 2010). At least in human neural progenitor cells, a global DNA hypomethylation by deletion of DNMT1 leads to activation of evolutionarily young hominoid-specific LINE-1 elements while the older L1s remain silent. Accordingly, activated L1s provide alternative promoter activity for many protein-coding genes that are relevant for neuronal functions. This shows that evolutionarily young L1-specific elements are controlled by a DNA methylation pattern (Jonsson et al., 2019). This situation could be provoked in AD brain by dysregulation of LINE-1 elements.

A recent study showed upregulation of the histone demethylase KDM4B in AD brains (Park et al., 2019). This histone demethylase had previously been identified to promote LINE-1 expression and enhance LINE-1 copy number and retrotransposition efficacy, while its depletion reduces LINE-1 expression (Xiang et al., 2019). In addition, SIRT6, a histone deacetylase and powerful repressor of L1-activity by ribosylating KAP1 (van Meter et al., 2014), a nuclear co-repressor protein of LINE-1 (Rowe et al., 2010; Castro-Diaz et al., 2014), is reduced in AD (Kaluski et al., 2017), which could further contribute to the activation of LINE-1.

Many more mutated APP-RNA variants were detected in single neuronal nuclei derived from prefrontal cortices of sporadic AD brains than in control brains (Lee et al., 2018). Some of these APP sequences showed intra-exonic junctions, and some even retained coding potential. Their presence in gencDNA might contribute to manifest sporadic AD cases. Since neurons are able to deliver linear and circular RNAs through exosome-dependent mechanisms (Liu et al., 2019), the propagation of mutated APP RNA transcripts to neighboring cells with the potential to be inserted as gencDNA by LINE-1 elements should be considered as a potential basis for pathology-spreading. Moreover, released extracellular vesicles can also mediate the horizontal transfer of active LINE-1 retrotransposons from one cell to another (Kawamura et al., 2019). In summary, a complete exchange of LINE-1 elements and trans RNAs like a tool kit seems possible.

## Therapeutic Implications

If the proposed mechanism is indeed instrumental to the AD pathomechanism, a moderate influence on LINE-1 activity might ameliorate the deleterious insertion of AD-related and mutated APP transcripts. The inhibition of reverse transcriptase activity, as already proposed elsewhere (Lee and Chun, 2019), might thus be a promising approach. Still, RT inhibitors would not prevent the generation of mutated APP mRNAs or their fragments. Moreover, since LINE-1 activity is necessary for memory formation, inhibition of RT would have potentially serious side effects that need to be considered.

Thus, it is necessary to accumulate more detailed knowledge of these mechanisms before any interferences regarding this mechanism can be envisaged.

## Author Contributions

UU and TA wrote the manuscript.

## Conflict of Interest

The authors declare that the research was conducted in the absence of any commercial or financial relationships that could be construed as a potential conflict of interest.

## Footnotes

1 http://www.bioinfo-cbs.org/igd
